# In Vitro Antioxidant Activity of Idebenone Derivative-Loaded Solid Lipid Nanoparticles

**DOI:** 10.3390/molecules22060887

**Published:** 2017-05-27

**Authors:** Lucia Montenegro, Maria N. Modica, Loredana Salerno, Anna Maria Panico, Lucia Crascì, Giovanni Puglisi, Giuseppe Romeo

**Affiliations:** Department of Drug Sciences, University of Catania, V. le A. Doria 6, 95125 Catania, Italy; mmodica@unict.it (M.N.M.); l.salerno@unict.it (L.S.); panico@unict.it (A.M.P.); luciacrasci@alice.it (L.C.); puglisig@unict.it (G.P.); gromeo@unict.it (G.R.)

**Keywords:** idebenone, lipoic acid, trolox, idebenone ester derivatives, antioxidant activity, ORAC

## Abstract

Idebenone (IDE) has been proposed for the treatment of neurodegenerative diseases involving mitochondria dysfunctions. Unfortunately, to date, IDE therapeutic treatments have not been as successful as expected. To improve IDE efficacy, in this work we describe a two-step approach: (1) synthesis of IDE ester derivatives by covalent linking IDE to other two antioxidants, trolox (IDETRL) and lipoic acid (IDELIP), to obtain a synergic effect; (2) loading of IDE, IDETRL, or IDELIP into solid lipid nanoparticles (SLN) to improve IDE and its esters’ water solubility while increasing and prolonging their antioxidant activity. IDE and its derivatives loaded SLN showed good physico-chemical and technological properties (spherical shape, mean particle sizes 23–25 nm, single peak in the size distribution, ζ potential values −1.76/−2.89 mV, and good stability at room temperature). In vitro antioxidant activity of these SLN was evaluated in comparison with free drugs by means of oxygen radical absorbance capacity (ORAC) test. IDETRL and IDELIP showed a greater antioxidant activity than IDE and encapsulation of IDE and its derivatives into SLN was able to prolong their antioxidant activity. These results suggest that loading IDETRL and IDELIP into SLN could be a useful strategy to improve IDE efficacy.

## 1. Introduction

**Figure 1:**
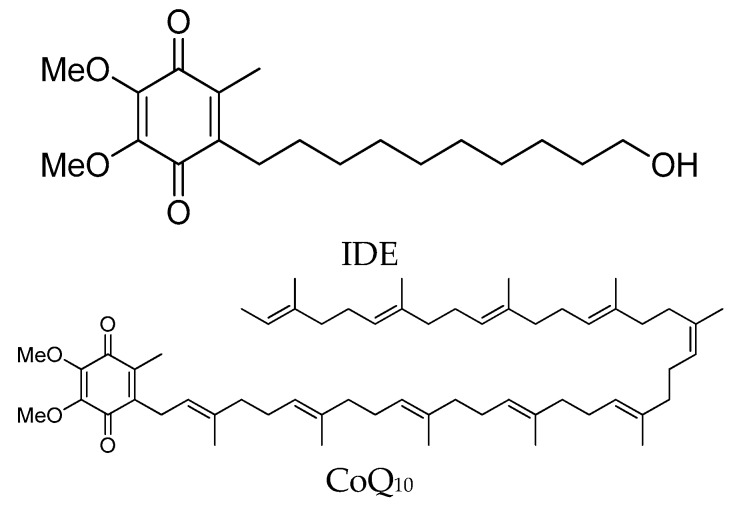
Chemical structures of IDE and CoQ_10_.

In 1986, Takeda Company Ltd. launched idebenone (IDE, Figure 1), a newly-developed potent antioxidant, for the treatment of emotional disturbances associated with cerebrovascular diseases [1]. To date, the actual mechanisms of the therapeutic effects of this synthetic analogue of coenzyme Q_10_ (CoQ_10_, Figure 1) are still under debate [2]. Several studies highlighted IDE’s ability to inhibit lipid peroxidation and to protect cell membranes and mitochondria from oxidative stress [3,4]. Being less lipophilic than CoQ_10_, IDE is supposed to localize at the surface of the mitochondrial inner membrane, whereas CoQ_10_ localization is assumed to be inside the membrane [5]. Therefore, at mitochondrial membrane level, CoQ_10_ could be responsible of lipoperoxide radicals scavenging while IDE could be active against hydrophilic superoxide radicals [5,6].

Data evidencing that IDE can transport electrons within the mitochondrial respiratory chain from complexes I and II to complex III [7,8], and directly from the cytosol to complex III [9], have prompted its use in the treatment of mitochondrial respiratory chain diseases [6,10,11,12]. However, several clinical trials on IDE beneficial effects in the treatment of neurodegenerative diseases, whose pathophysiological processes involve mitochondrial dysfunctions, have provided conflicting results. Di Prospero et al. [13] enrolled Friedreich’s ataxia (FRDA) patients who were administered different IDE doses in a six-month, randomized, double-blind, and placebo-controlled study. The authors observed a strong correlation between IDE dosage and improvement of neurological function, thus suggesting the need of using high-dose IDE to ameliorate FRDA neurological symptoms. On the contrary, two multi-center phase III randomized, placebo-controlled trials (IONIA and MICONOS) on large cohorts of FRDA patients evidenced the failure of high-dose IDE in improving the neurological function of adult and pediatric patients [14,15]. However, these studies confirmed the efficacy of IDE in the reduction of hypertrophic cardiomyopathy associated with FRDA and the safety of this drug at high doses, already reported in other clinical trials [16,17]. At present, IDE is under clinical investigation in the USA for the treatment of other pathologies associated with mitochondrial dysfunctions, such as Leber’s hereditary optic neuropathy (LHON), Duchenne muscular dystrophy (DMD), and primary progressive multiple sclerosis (PPMS) (clinicaltrials.gov NCT02771379; clinicaltrials.gov NCT02814019; clinicaltrials.gov NCT01854359) while, in 2015, it was approved by the EMA (European Medicines Agency) for the treatment of LHON patients.

In addition to clinical studies on IDE, in recent years different strategies have been attempted to use CoQ_10_ in the treatment of neurodegenerative diseases. Two pilot studies, investigating the effects of co-administering CoQ_10_ and vitamin E, provided data that have evidenced a slowing of the progression of some neurological symptoms, along with an improvement of both cardiac and skeletal muscle function [18,19]. Another approach aimed at improving CoQ_10_ efficacy by its conjugation to the triphenylphosphonium cation [20,21]. The resulting compound, MitoQ, was able to accumulate selectively within mitochondria owing to its ease of passage through biological membranes, thus improving the protection of these organelles from oxidative stress [20]. Furthermore, MitoQ targeting to mitochondria resulted in an antioxidant activity several hundred-fold higher than that of IDE [21]. However, further studies are needed to demonstrate the clinical efficacy of MitoQ.

The design and synthesis of bi-functional molecules is a field extensively studied [22,23,24] and has the aim to obtain an improvement of pharmacological effect compared to a two-drug administration, a reduction of dosage and side effects, and a better patient compliance.

**Scheme 1:**
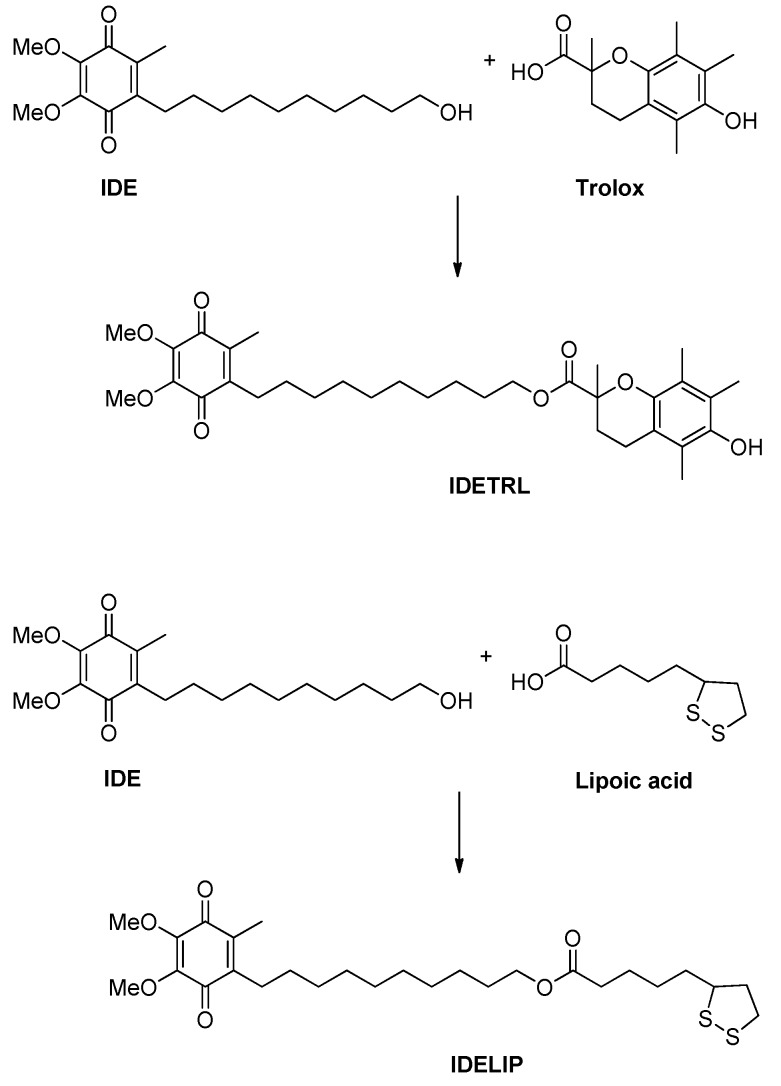
Reagents and conditions: DCC, 4-DMAP, dry CH_3_CN, 0 °C, then room temperature, 1.5 and 5 h under nitrogen.

In this work, to improve IDE efficacy, we synthesized two ester derivatives covalent linking IDE to trolox (IDETRL, Scheme 1) or lipoic acid (IDELIP, Scheme 1). Trolox is a cell-permeable analog of vitamin E with potent antioxidant properties, used to inhibit oxidative stress for biological or biochemical applications and as reference standard [25,26]. Lipoic acid, the coenzyme of mitochondrial pyruvate dehydrogenase and α-ketoglutarate dehydrogenase, shows strong mitochondrial antioxidant properties due to its ability to recycle other antioxidants (e.g., vitamin E and C, glutathione) and to scavenge both reactive oxygen species (ROS) and lipid peroxidation products [27]. Some studies on neurological outcomes, after lipoic acid administration in Alzheimer’s disease patients, evidenced reduction of memory loss and stabilization of cognitive function [28].

To date, all clinical trials on IDE have been carried out using IDE oral dosage forms, due to IDE poor water solubility, which prevents its parenteral administration in aqueous vehicles. Despite its rapid absorption after oral dosing, IDE bioavailability is poor owing to an extensive first-pass effect that could prevent the achievement of therapeutic plasma concentration, even at high doses [12].

Loading IDE into suitable drug delivery systems could improve its water solubility to allow its parenteral administration, thus, opening new perspectives in the clinical use of IDE.

In previous studies [29,30], we reported the feasibility of using IDE loaded solid lipid nanoparticles (SLN) as carriers for IDE delivery to the brain. Due to their small size, these nanoparticles could penetrate through the blood brain barrier (BBB) and escape reticuloendothelial system (RES) uptake, thus showing prolonged circulation into the blood. Furthermore, cytotoxicity studies evidenced their good tolerability and in vitro tests in cultures of astrocytes obtained from rat cerebral cortex showed their ability to inhibit completely ROS production.

IDE ester derivatives, synthesized in this work, were expected to be more lipophilic than IDE as estimated by calculated Log P performed by ACD/ChemSketch Freeware (Toronto, ON, Canada) [31] (Log P IDE 3.49 ± 1; Log P IDETRL 7.52 ± 1; Log P IDELIP 6.83 ± 1). Therefore, to improve their water solubility, we incorporated IDE and its ester derivatives in SLN and evaluated in vitro antioxidant activity of these SLN in comparison with free drugs by means of oxygen radical absorbance capacity (ORAC) test.

## 2. Results and Discussion

### 2.1. Solid Lipid Nanoparticle Characterization 

**Figure 2:**
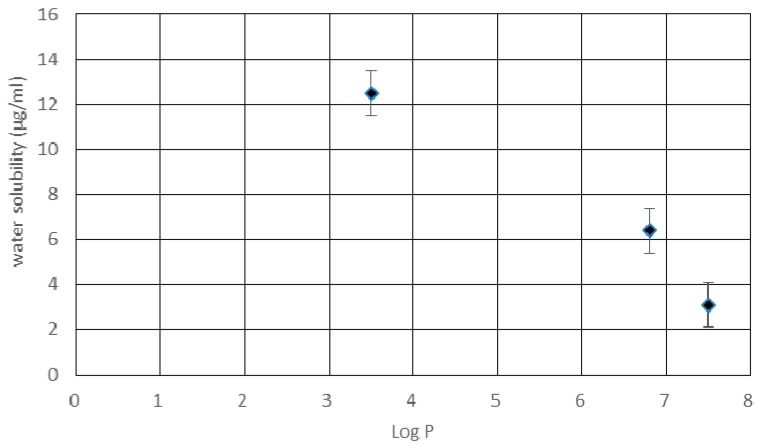
Relationship between Log P and water solubility of IDE (Log P 3.49), IDELIP (Log P 6.83), and IDETRL (Log P 7.52).

As expected from calculated Log P values, IDE esters were less water soluble than IDE (water solubility: IDE 12.5 µg/mL; IDETRL 3.1 µg/mL; IDELIP 6.4 µg/mL). IDE water solubility determined in this work was in same order of magnitude of that reported in literature [32]. Plotting IDE and its esters water solubility vs. their Log P values, we observed a decrease of water solubility when increasing Log P values (Figure 2). Due to few data points, a significant correlation analysis could not be performed. However, these results suggest the need to design IDE derivatives with Log P values lower than that of IDE to achieve an improvement of solubility in aqueous vehicles. 

To overcome the drawback of poor water solubility and to improve and prolong the antioxidant activity of IDE and its esters, we incorporated these compounds into SLN.

**Figure 3:**
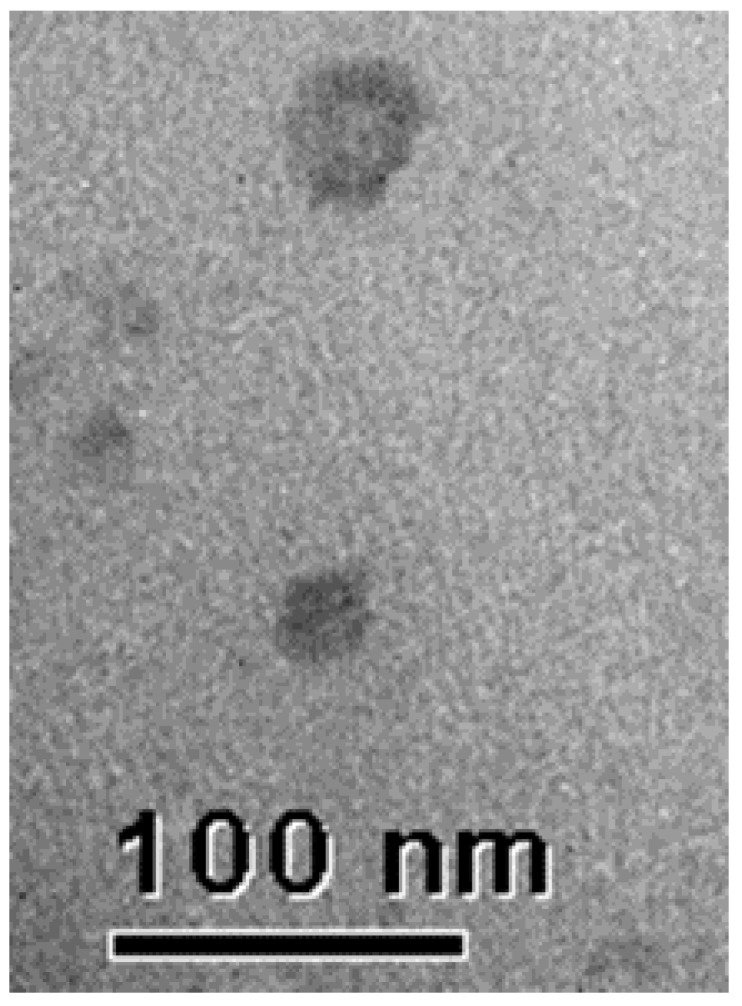
TEM image of IDE loaded SLN.

All the SLN were prepared using the phase inversion temperature (PIT) method that provided nanoparticles with good physico-chemical and technological properties as shown in previous studies [29,30,33]. Figure 3 reports a typical transmission electron microscopy (TEM) image of these SLN, showing spherical particles with a narrow dimensional distribution. 

As reported in Table 1, all the SLN showed mean particle sizes in the range of 23–25 nm, a single peak in size distribution (polydispersity index < 0.300) and slightly negative ζ potential values (−1.76/−2.89 mV). Loading IDE, IDETRL, IDELIP, or a mixture of IDE and trolox or lipoic acid into these carriers did not significantly affect their morphology and their physico-chemical parameters. Despite their low ζ potential values, all the SLN were stable after storage for two months at room temperature as no changes of particle size, polydispersity index, and ζ potential values were observed (data not shown). Izquierdo et al. [34] reported that the HLB (hydrophilic lipophilic balance) temperature (or PIT temperature) could be regarded as predictive of the stability of emulsifying based systems because an increase of the PIT temperature provides an increase of the formulation stability. The incorporation of IDE or its derivatives into SLN resulted in an increase of PIT values compared to unloaded SLN, thus suggesting that high PIT values could play a key role in determining the stability of these drug delivery systems, in addition to ζ potential values.

In a previous work [33], IDE loading capacity in the same type of SLN was found to be 1.1% *w*/*w* and was expressed as the maximum amount of drug that could be incorporated into the nanoparticles leading to a clear vehicle with no sign of precipitation. As reported in the literature [35], very lipophilic compounds are supposed to be completely incorporated into the SLN when the formulation is clear; otherwise, the unloaded drug would give rise to a precipitate or at least to a turbid system.

In this work, using the same method described above, we determined that IDETRL and IDELIP loading capacity was 0.5% *w*/*w* and, therefore, it was lower than that of IDE. These results could be attributed to IDETRL and IDELIP physico-chemical properties (higher lipophilicity and lower water solubility than IDE) that could hinder their incorporation into the nanoparticles. As to compare in vitro antioxidant activity of loaded SLN we needed SLN containing the same percentage of active compounds, we incorporated into SLN 0.5% *w*/*w* of IDE and IDE esters, despite a greater IDE loading capacity. In this way, we were able to obtain aqueous formulations containing an amount of active compound much greater than that corresponding to its saturation concentration. The increase of IDE, IDETRL, and IDELIP water solubility due to their loading into SLN was 400-fold for IDE, 780-fold for IDETRL, and 1600-fold for IDELIP. Owing to their very small sizes (23–25 nm), these SLN could be supposed to escape easily the RES uptake and to permeate across the BBB. In a previous work evaluating in vitro transport of similar IDE loaded SLN across a model of BBB consisting of MDCKII-MDR1 cell monolayers, we evidenced that IDE could permeate via a transcellular pathway [30]. Therefore, further in vitro and in vivo studies are ongoing to investigate IDE and its derivative loaded SLN tolerability and their pharmacokinetic and pharmacodynamic profile after parenteral administration in suitable animal models. As IDETRL and IDELIP are ester derivatives, they could be hydrolyzed after their release from SLN. Therefore, further in vitro and in vivo studies will include the evaluation of the hydrolysis rate of these derivatives at cellular level and in biological fluids.

### 2.2. In Vitro Antioxidant Activity

ROS such as superoxide anion (O_2_^•−^), hydroxyl (^•^OH), peroxyl (ROO^•^), and alkoxyl radicals (RO^•^), hydrogen peroxide (H_2_O_2_), and singlet oxygen (^1^O_2_) may attack biological macromolecules, giving rise to protein, lipid, and DNA damages. Organisms have developed complex antioxidant systems to protect themselves from oxidative stress. However, when ROS production overwhelms the physiological defense mechanisms, an oxidative stress occurs. Therefore, ROS are supposed to be involved in several disease states, including inflammation, premature aging, cancer, diabetes, atherosclerosis, osteoarthritic, and neurodegenerative diseases. At present, supplementation with antioxidants by different administration routes (oral, parenteral, topical) is regarded as a promising strategy to counteract ROS biological damages. Therefore, in recent years, several methods have been developed to assess in vitro and in vivo antioxidant potential of molecules whose activity could be beneficial in the treatment of disorders involving oxidative stress [36,37,38].

In this work, to evaluate the antioxidant activity of IDE and its derivatives and of SLN containing these active compounds, we used the ORAC assay as in a previous study this method proved to be suitable to assess the antioxidant activity of IDE loaded SLN [39]. Furthermore, other authors used the ORAC assay to evaluate in vitro antioxidant activity of derivatives of antioxidant molecules [40].

This sensitive assay is based on the detection of the decrease of fluorescein (FL) fluorescence emission due to the chemical damage caused by peroxyl radicals, generated in situ by the decomposition of the initiator AAPH (2,2-azobis(2-amidinopropane)-dihydrochloride) [41]. The capacity of tested compounds to scavenge peroxyl radicals was evaluated in comparison with trolox as standard and the results were expressed as trolox equivalent for µM (TE/µM) of samples.

As evidenced in Table 2, IDE and its ester derivatives IDETRL and IDELIP showed an antioxidant activity greater than that of the reference standard trolox. Lipoic acid, used to obtain the ester IDELIP, exhibited an ORAC value lower than that of trolox, thus confirming lipoic acid poor scavenging activity against the peroxyl radical generated in the ORAC test, as already reported by others [42]. It is worth noting that the ester IDELIP and the physical mixture IDE/LIP had similar ORAC values and both were more active than IDE. Therefore, an additive activity between IDE and lipoic acid (LIP) was observed, regardless of the presence of an ester linkage between these molecules. On the contrary, esterifying IDE with TRL led to a synergic effect while the physical mixture IDE/TRL showed the same ORAC value of IDE alone. Further studies are planned to elucidate the mechanisms that led to the different behavior of IDELIP and IDETRL and their physical mixtures.

Unloaded SLN showed an ORAC value similar to trolox, which could be due to the presence of oleic residues in SLN components, oleth-20 (ORAC value 0.78) and glyceryl oleate (GO, ORAC value 0.85), as other authors reported a moderate antioxidant activity for polyunsaturated triglycerides such as triolein [43]. The ORAC test was not performed on cetyl palmitate as no antioxidant activity could be attributed to this molecule, owing to its chemical structure (saturated long chain acyl ester). 

Loading IDE, IDETRL, IDELIP, and the physical mixtures IDE/TRL and IDE/LIP into SLN, ORAC values similar to those of free active compounds were observed. These results suggest that the use of SLN as carriers did not significantly increase ORAC values of the compounds under investigation. 

**Figure 4:**
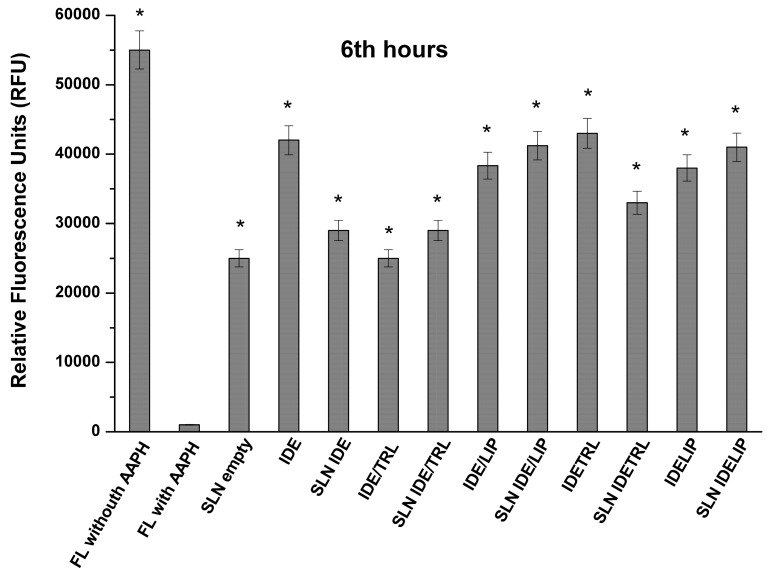
Antioxidant efficiency of IDE, IDETRL, IDELIP, and of the physical mixtures IDE/TRL and IDE/LIP, free or loaded into SLN, at the 6th hour. Data represent the mean of three independent experiments ± SD. * *p* < 0.05 with respect to the positive control.

**Figure 5:**
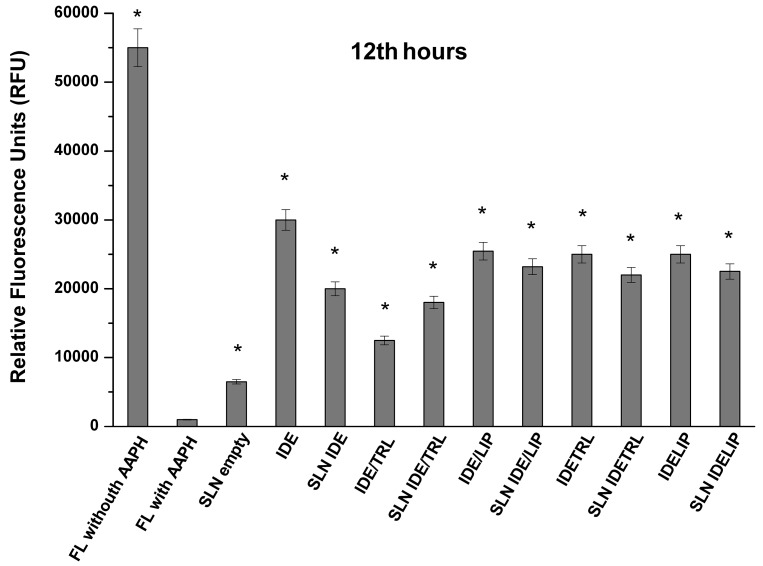
Antioxidant efficiency of IDE, IDETRL, IDELIP, and of the physical mixtures IDE/TRL and IDE/LIP, free or loaded into SLN, at the 12th hour. Data represent the mean of three independent experiments ± SD. * *p* < 0.05 with respect to the positive control.

**Figure 6:**
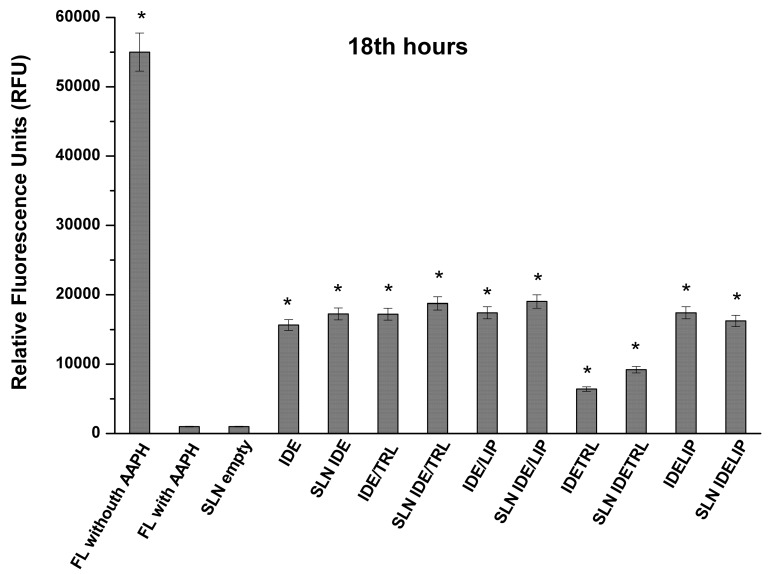
Antioxidant efficiency of IDE, IDETRL, IDELIP, and of the physical mixtures IDE/TRL and IDE/LIP, free or loaded into SLN, at the 18th hour. Data represent the mean of three independent experiments ± SD. * *p* < 0.05 with respect to positive control.

**Figure 7:**
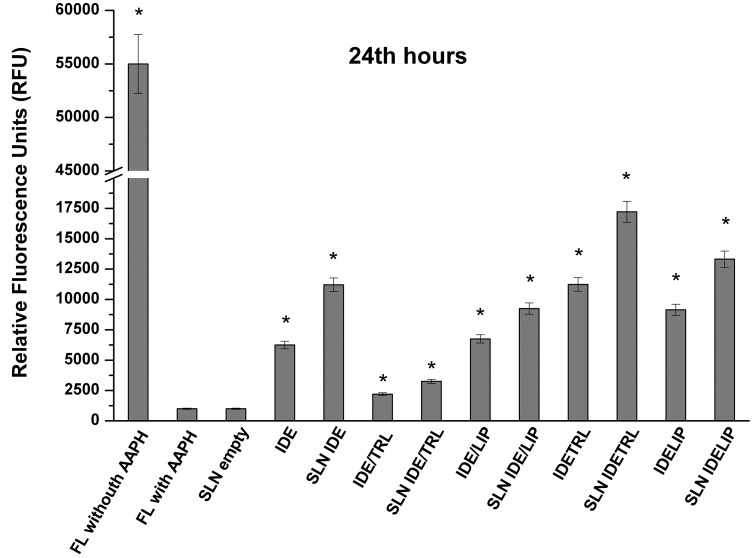
Antioxidant efficiency of IDE, IDETRL, IDELIP, and of the physical mixtures IDE/TRL and IDE/LIP, free or loaded into SLN, at the 24th hour. Data represent the mean of three independent experiments ± SD. * *p* < 0.05 with respect to the positive control.

**Figure 8:**
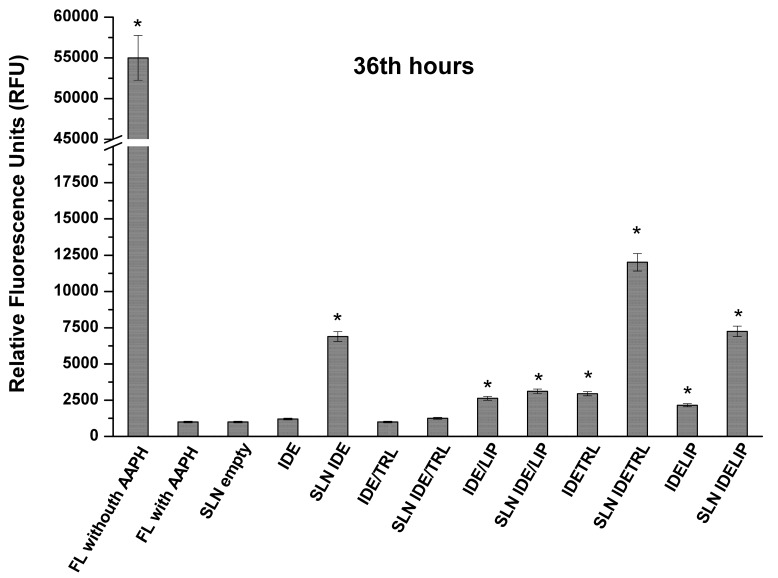
Antioxidant efficiency of IDE, IDETRL, IDELIP, and of the physical mixtures IDE/TRL and IDE/LIP, free or loaded into SLN, at the 36th hour. Data represent the mean of three independent experiments ± SD. * *p* < 0.05 with respect to the positive control.

To evaluate SLN ability to prolong the antioxidant effect of the incorporated compounds, we investigated the antioxidant efficiency as function of time, using a modified ORAC assay. Sample ability to inhibit the free radical damage of the fluorescent probe was expressed in terms of preservation of the fluorescent signal. In Figure 4, Figure 5, Figure 6, Figure 7 and Figure 8, we reported the ability of IDE, IDETRL, IDELIP, and of the physical mixtures IDE/TRL and IDE/LIP, free or loaded into SLN, to maintain the fluorescence signal, expressed as relative fluorescence unit (RFU), at different time intervals (6, 12, 18, and 24 h). Figure 4, Figure 5, Figure 6, Figure 7 and Figure 8 show that the fluorescence of a 10.0 nM FL solution, in the absence of AAPH, was preserved over 36 h of incubation.

After 6 h of incubation (Figure 4), all of the samples under investigation were able to prevent FL damage induced by AAPH, although to a different extent. The results after 12 h of incubation (Figure 5) evidenced that all the samples showed a decreased antioxidant efficiency, but the activity trend was similar to that observed after 6 h of incubation. A further reduction of the ability to prevent FL damage was observed after 18 h of incubation (Figure 6) and a complete quenching of FL signal was observed for unloaded SLN. It is worth noting that after 24 h and 36 h of incubation (Figure 7 and Figure 8, respectively), all the samples containing free active compounds were less effective than the corresponding samples containing SLN. In particular, after 36 h the antioxidant efficiency of SLN loaded with IDE and its esters was in the order SLN IDETRL (12012 RFU) > SLN IDELIP (7250 RFU) > SLN IDE (6890 RFU), thus suggesting that their effectiveness was related to the ORAC value of the incorporated active compound. Furthermore, these results support the usefulness of loading antioxidants into SLN to prolong their activity, as already reported by others [44,45,46]. 

Being IDETRL and IDELIP more lipophilic than IDE, their location at mitochondrial level could be different from that of IDE as well as their interaction with the respiratory chain. Furthermore, loading IDE and its derivatives into SLN could affect release, distribution, and disposition of these molecules inside the cells and into the different cellular compartments. Therefore, further studies are needed to elucidate the mechanisms involved in the antioxidant activity of IDE and its derivatives loaded SLN at the cellular level.

## 3. Materials and Methods

### 3.1. Materials

Polyoxyethylene-20-oleyl ether (Brij 98^®^, Oleth-20) was bought from ACEF (Fiorenzuola D’Arda, Piacenza, Italy). Glyceryl oleate (Tegin O^®^, GO) was obtained from Th. Goldschmidt Ag (Milan, Italy). Cetyl palmitate (Cutina CP^®^, CP) was a gift from Basf (Ludwigshafen, Germany). Methylchloroisothiazolinone and methylisothiazolinone (Kathon CG^®^), and imidazolidinyl urea (Gram 1^®^) were kindly supplied by Sinerga (Milan, Italy). 2,2′-Azobis(2-methylpropionamidine) dihydrochloride (AAPH) and fluorescin (FL) were bought from Sigma-Aldrich srl (Milan, Italy).

All chemicals and solvents used to synthesize Idebenone derivatives were reagent grade and were purchased: trolox (TRL), lipoic acid (LIP), *N*,*N*-dicyclohexylcarbodiimide (DCC), dry acetonitrile (CH_3_CN), 4-dimethylaminopyridine (4-DMAP), ethyl acetate (EtOAc), and hydrochloric acid (HCl) from Sigma-Aldrich srl (Milan, Italy); idebenone (IDE) from Carbosynth Limited (Berkshire, UK).

### 3.2. Chemistry

The synthesis of esters IDETRL and IDELIP was reported in Scheme 1. Commercially-available idebenone and trolox or lipoic acid were reacted to obtain esters IDETRL and IDELIP, respectively, in the presence of 4-DMAP and DCC in dry acetonitrile as solvent. This procedure had already been adopted for the preparation of IDETRL, known as Fe-Aox29 [47]; in this case, some slight modifications were used.

Infrared spectra were recorded on a Perkin Elmer series FTIR 1600 spectrometer (Milan, Italy) in NaBr disks. ^1^H-NMR spectra were performed on a Varian Inova Unity 200 spectrometer (200 MHz) (Milan, Italy) in DMSO-*d*_6_ or CDCl_3_ solution. Chemical shifts are given in δ values (ppm), using tetramethylsilane as the internal standard; coupling constants (*J*) are given in Hz. Signal multiplicities are characterized as s (singlet), t (triplet), and m (multiplet). Reaction progress and purity of all the synthesized compounds were checked on TLC (an aluminum sheet coated with silica gel 60 F_254_, Merck, Darmstadt, Germany) and visualized by UV light at 254 and 366 nm as wavelength). Purification of synthesized compounds by flash column chromatography was performed using Merck silica gel (0.040–0.063 mm).

### 3.3. Synthesis of IDE Derivatives

#### 3.3.1. 3,4-Dihydro-6-hydroxy-2,5,7,8-tetramethyl-2*H*-1-benzopyran-2-carboxylic acid 10-(4,5-dimethoxy-2-methyl-3,6-dioxo-1,4-cyclohexadien-1-yl)decyl ester (IDETRL)

Idebenone (676 mg, 1.99 mmol) and 4-DMAP (25 mg, 0.20 mmol) were added to a solution of trolox (500 mg, 1.99) in dry CH_3_CN (25 mL). To the mixture was added, drop-wise at 0 °C, a suspension of DCC (620 mg, 3.00 mmol) in dry CH_3_CN (8.5 mL). Then the mixture was stirred under nitrogen at room temperature for 1.5 h. The obtained precipitate of dicyclohexylurea was eliminated by filtration and the solvent was evaporated in vacuum to dryness. The residue was dissolved in ethyl acetate, washed with 0.5 N HCl (25 mL × 2), H_2_O (25 mL), and brine (25 mL). The organic phase was dried on anhydrous sodium sulphate, filtered, and evaporated in vacuum to dryness. The obtained oil was purified by flash column chromatography using ethyl acetate as eluent to give IDETRL as a pure orange oil (940 mg, 82%).

IR (neat, selected lines) cm^−1^ 2929, 2854, 1728, 1651, 1610, 1455, 1263, 1204, 1139, 1111. ^1^H-NMR (CDCl_3_) δ 1.08–1.58 (m, 16H, 8 CH_2_), 1.59 (s, 3H, CH_3_, chromane), 1.78–2.00 (m, 1H, CH_a_H_b_CH_2_C=C), 2.01 (s, 3H, CH_3_, quinone), 2.06 (s, 3H, CH_3_, Ar), 2.15 (s, 3H, CH_3_, Ar), 2.17 (s, 3H, CH_3_, Ar), 2.38–2.70 (m, 5H, CH_2_CC=O + CH_2_C=C + CH_a_H_b_CH_2_C=C), 3.85–4.18 (m, 8H, OCH_3_ + OCH_3_ + CH_2_O). C_33_H_46_O_8_.

#### 3.3.2. 5-(1,2-Dithiolan-3-yl)pentanoic acid 10-(4,5-dimethoxy-2-methyl-3,6-dioxo-1,4-cyclohexadien-1-yl)decyl ester (IDELIP)

Idebenone (500 mg, 1.47 mmol) and 4-DMAP (17.5 mg, 0.14 mmol) were added to a solution of lipoic acid (332 mg; 1.61) in dry CH_3_CN (20 mL). To the mixture was added, drop-wise at 0 °C, a suspension of DCC (445 mg, 2.16 mmol) in dry CH_3_CN (10 mL). Then the mixture was stirred under nitrogen at room temperature for 5 h. The obtained precipitate of dicyclohexylurea was eliminated by filtration and the solvent was evaporated in vacuum to dryness. The residue was dissolved in ethyl ether, washed with 0.5 N HCl (25 mL × 2), H_2_O (25 mL), and brine (25 mL). The organic phase was dried on anhydrous sodium sulphate, filtered, and evaporated in vacuum to dryness. The obtained oil was purified by flash column chromatography using a mixture of cyclohexane/ethyl acetate (8:2, *v*/*v*) as eluent to give IDELIP as a pure orange oil (400 mg, 51%).

IR (neat, selected lines) cm^−1^ 2927, 2853, 1733, 1648, 1610, 1458, 1265, 1204, 1068. ^1^H-NMR (DMSO-*d*_6_) δ 1.08–1.45 (m, 16H, 8 CH_2_), 1.45–1.75 (m, 6H, 3 CH_2_), 1.75–1.90 (m, 1H, CH_a_H_b_CHS, dithiolane), 1.92 (s, 3H, CH_3_), 2.29 (t, *J* = 7.4 Hz, 2H, CH_2_CO), 2.35–2.49 (m, 3H, CH_a_H_b_CHS, dithiolane + CH_2_CC=O), 3.05–3.35 (m, 2H, CH_2_S), 3.50–3.68 (m, 1H, CH_2_CHS), 3.87 (s, 6H, OCH_3_ + OCH_3_), 3.99 (t, *J* = 6.4 Hz, 2H, CH_2_O). C_27_H_42_O_6_S_2_.

### 3.4. IDE and Its Derivatives’ Water Solubility

IDE and its derivatives’ water solubility was determined in duplicate by stirring an excess of each compound in deionized water with a magnetic stirrer at room temperature, for 24 h. Thereafter, the resulting suspensions were filtered and the concentration of the compound in its saturated solution was determined spectrophotometrically (Shimadzu mod. UV-1601, Milan, Italy) at 278 nm for IDE, at 285 nm for IDETRL and at 280 nm for IDELIP. A standard working curve was constructed from a known concentration of each compound in deionized water. The sensitivity of the assay was 0.5 µg/mL for IDE and 1 µg/mL for IDETRL and IDELIP.

### 3.5. Solid Lipid Nanoparticle Preparation

All SLN were prepared using the phase inversion temperature (PIT) method, according to a procedure previously described [29]. The oil phase consisted of CP, oleth-20, GO, and IDE or its derivatives or a physical mixture of IDE and TRL or LIP. The amount of IDE, TRL, and LIP in the physical mixture were calculated considering the percentage (*w*/*w*) of each compound contained in a mole of IDETRL or IDELIP. The percentages (*w*/*w*) of each compound used to prepare such SLN are reported in Table 3. After separately heating at ~90 °C the aqueous phase (deionized water containing 0.35% *w*/*w* Gram 1^®^ and 0.05% *w*/*w* Kathon CG^®^ as preservatives) and the oil phase, the first was added drop by drop, at constant temperature and under mixing, to the oil phase. Then, the turbid mixture was cooled to room temperature under continuous stirring. At the phase inversion temperature (PIT), the mixture turned into clear and PIT values were recorded using a conductivity meter (model 525, Crison, Modena, Italy). A thin layer chromatography (TLC) analysis confirmed that no degradation of IDE or its derivatives occurred under these conditions. SLN samples were stored at room temperature in airtight vials sheltered from the light.

### 3.6. Transmission Electron Microscopy (TEM)

Negative-staining electron microscopy was performed on 5 µL of each SLN samples, which were placed on a 200-mesh formvar copper grid (TAAB Laboratories Equipment, Berks, UK). After sample absorption, the surplus was removed by filter paper and a drop of 2% (*w*/*v*) aqueous solution of uranyl acetate was added over 2 min. After removing the surplus, the sample was allowed to dry at room temperature prior to its analysis with a transmission electron microscope (model JEM 2010, Jeol, Peabody, MA, USA), operating at an acceleration voltage of 200 KV.

### 3.7. Photon Correlation Spectroscopy (PCS)

For freshly-prepared SLN, the mean particle size and the size distribution were determined, using a Zetasizer Nano ZS90 (Malvern Instruments, Malvern, UK), by scattering light at 90 °C using a 4 mW laser diode operating at 670 nm. Samples were analyzed after dilution (1:1, sample/distilled water) and equilibration to 25 °C for 2 min prior to the analysis. The results were expressed as mean ± S.D. of three replicates of two separate preparations. 

The determination of the ζ-potential was performed using the technique of laser Doppler velocimetry using a Zetasizer Nano ZS 90 after dilution with KCl 1 mM (pH 7.0), according to a procedure already reported [30].

### 3.8. Oxygen Radical Absorbance Capacity (ORAC) Assay

The antioxidant activity of IDE and its derivatives loaded SLN was evaluated in vitro using the oxygen absorbance capacity (ORAC) assay, which was performed as previously reported by Cao et al. [48]. The measurements were carried out using a VICTOR Wallac 1420 Multilabel Counters fluorimeter (Perkin Elmer, Boston, MA, USA) with a fluorescence filter (excitation 540 nm, emission 570 nm). Fluorescein (FL) solution (10 nM) was the fluorescence probe and the target molecule for free radical attack by AAPH (100 mM), a peroxyl radical generator. The reaction was conducted at 37 °C and at pH 7.0, with trolox (12.5 μM) as the control standard and phosphate buffer as the blank. IDE, IDETRL, IDELIP, LIP, and the physical mixture (in the same ratio loaded into SLN) IDE/TRL and IDE/LIP were diluted at 12.5 μM with ethanol. SLN components (oleth-20 and GO), loaded and unloaded SLN were diluted at 12.5 μM with phosphate buffer before analysis. The fluorescence was recorded every 2 min after addition of AAPH. All measurements were expressed relatively to the initial reading. One blank, one standard, and all samples were analyzed at the same time. Each measure was repeated at least three times. The ORAC value refers to the net protection area under the quenching curve of FL in the presence of an antioxidant. The results (ORAC values) were calculated and were expressed using trolox equivalents (TE) for μM of sample (TE/μM) according to the following equation:

ORAC units (TE/μM) = K (Ssample−Sblank)/(Strolox−Sblank)
(1)

where K is a sample dilution factor and S is the area under the fluorescence decay curve of the sample, trolox, or blank. This area was calculated with Origin^®^7 (OriginLab Corporation, Northampton, MA, USA).

### 3.9. Antioxidant Efficiency

To qualitatively determine SLN’s ability to maintain or prolong the antioxidant activity of IDE and its derivatives, a modified ORAC assay was used. Samples (100 µL) of free or SLN-loaded IDE, IDETRL, IDELIP, IDE/TRL, and IDE/LIP were placed in 96-well tissue culture plates. Wells bordering the outermost edge of the plate were not used because they have been found to distort fluorescence measurements as well as to introduce temperature variance in the assay. AAPH (25 µL, 100 mM) was used as a radical initiator to generate free radicals at a constant rate. FL (150 µL, 10.0 nM) was used as a probe to assess the antioxidant activity. A positive control (FL solution containing AAPH), a negative control (FL solution without AAPH), unloaded SLN and loaded SLN diluted with phosphate buffer, and free active compounds (IDE, IDE/TRL, IDE/LIP, IDETRL, IDELIP) diluted in ethanol at the same concentration reported in Table 1, were run simultaneously. A timer was started upon introduction of the free radical generator and the plate was stored in the dark at 37 °C. At each specified time point (6, 12, 18, 24, and 36 h), the relative fluorescence units (RFU) of each sample were measured (excitation 540 nm, emission 570 nm) using a Victor Wallac 1420 Multilabel Counter fluorimeter (Perkin Elmer, Boston, MA, USA) and were plotted as a function of time [49].

## Figures and Tables

**Table 1:** Mean size (size), polydispersity index (PDI), zeta potential (ζ), and phase inversion temperature (PIT) of unloaded and drug loaded SLN.

SLN	Size ± S.D. (nm)	PDI ± S.D.	ζ ± S.D. (mV)	PIT (°C)
B (empty)	23.72 ± 0.08	0.101 ± 0.010	−1.76 ± 0.48	65
IDE	25.72 ± 0.01	0.106 ± 0.018	−2.89 ± 0.86	70
IDETRL	25.09 ± 0.07	0.096 ± 0.011	−2.03 ± 0.61	70
IDELIP	23.62 ± 0.06	0.081 ± 0.012	−2.22 ± 0.77	72
IDE/TRL	24.55 ± 0.05	0.094 ± 0.014	−1.90 ± 0.81	71
IDE/LIP	23.98 ± 0.08	0.112 ± 0.011	2.35 ± 0.93	72

**Table 2:** Antioxidant activity expressed as ORAC units of IDE, IDE derivatives, SLN components, (Oleth-20, GO, LIP), unloaded and loaded SLN.

Compounds	ORAC Units ^a^	SLN	ORAC Units ^a^
Oleth-20	0.78 ± 0.03 *	SLN B (empty)	1.19 ± 0.18
GO	0.85 ± 0.02 *		
LIP	0.63 ± 0.03*		
IDE	1.48 ± 0.15 *	SLN IDE	1.79 ± 0.25 *
IDE/TRL	1.49 ± 0.14 *	SLN IDE/TRL	1.54 ± 0.09 *
IDE/LIP	2.22 ± 0.25 *	SLN IDE/LIP	2.65 ± 0.31 *
IDETRL	3.94 ± 0.24 *	SLN IDETRL	4.32 ± 0.12 *
IDELIP	2.18 ± 0.18 *	SLN IDELIP	2.13 ± 0.23 *

^a^ Expressed as trolox equivalents for µM of sample (trolox = 1.00 ± 0.02 ORAC units). Data represent the mean of three independent experiments ± SD. * *p* < 0.05 versus Trolox.

**Table 3:** Composition of the oil phase of unloaded and drug loaded SLN ^a^.

SLN Code	Ingredient (% *w*/*w*)							
	Oleth-20	GO	CP	IDE	IDETRL	IDELIP	TRL	LIP
B (empty)	8.70	4.40	5.00	---	---	---	---	---
IDE	8.70	4.40	5.00	0.50	---	---	---	---
IDETRL	8.70	4.40	5.00	---	0.50	---	---	---
IDELIP	8.70	4.40	5.00	---	---	0.50	---	---
IDE/TRL	8.70	4.40	5.00	0.30	---	---	0.20	---
IDE/LIP	8.70	4.40	5.00	0.32	---	---	---	0.18

^a^ SLN water phase q.b. 100.00.
